# Motivators and Barriers to Incorporating Climate Change-Related Health Risks in Environmental Health Impact Assessment

**DOI:** 10.3390/ijerph10031139

**Published:** 2013-03-22

**Authors:** Lyle R. Turner, Katarzyna Alderman, Des Connell, Shilu Tong

**Affiliations:** 1 School of Public Health and Social Work, Institute of Health and Biomedical Innovation, Queensland University of Technology, Victoria Park Road, Kelvin Grove, QLD 4059, Australia; E-Mail: lr.turner@qut.edu.au; 2 School of Population Health, University of Queensland, Herston Road, Herston, QLD 4006, Australia; E-Mail: k.alderman@uq.edu.au; 3 School of Environment, Griffith University, Nathan Campus, QLD 4111, Australia; E-Mail: d.connell@griffith.edu.au

**Keywords:** environmental health impact assessment, climate change, public policy, data linkage, health indicators, focus group

## Abstract

Climate change presents risks to health that must be addressed by both decision-makers and public health researchers. Within the application of Environmental Health Impact Assessment (EHIA), there have been few attempts to incorporate climate change-related health risks as an input to the framework. This study used a focus group design to examine the perceptions of government, industry and academic specialists about the suitability of assessing the health consequences of climate change within an EHIA framework. Practitioners expressed concern over a number of factors relating to the current EHIA methodology and the inclusion of climate change-related health risks. These concerns related to the broad scope of issues that would need to be considered, problems with identifying appropriate health indicators, the lack of relevant qualitative information that is currently incorporated in assessment and persistent issues surrounding stakeholder participation. It was suggested that improvements are needed in data collection processes, particularly in terms of adequate communication between environmental and health practitioners. Concerns were raised surrounding data privacy and usage, and how these could impact on the assessment process. These findings may provide guidance for government and industry bodies to improve the assessment of climate change-related health risks.

## 1. Introduction

Despite the complex relationships that exist between the environment and health, the assessment of related impacts has often been performed separately, with health impacts often neglected within traditional environmental impact assessment frameworks [1,2]. It has been recognised that to address this imbalance both must be assessed together [3]. Consequently, the use of Environmental Health Impact Assessment (EHIA) has become increasingly common, particularly to accommodate both direct and indirect linkages between the environment and health risks [4].

EHIA has been defined as an environmental impact assessment procedure with a health component included, formalising the process of assessing both direct and indirect health impacts of an environmental change, project, development, or policy [2]. In Australia, EHIA is often viewed as a process which combines both Environmental Impact Assessment and Health Impact Assessment when evaluating the impacts of a development, policy or plan on the environment and on health [5]. In the past, the impacts of health hazards related to environmental contamination, planning activities and policy have been included in assessments, with the range of potential applications implying that EHIAs must be flexible and have the capacity to incorporate a wide variety of issues [6,7].

It is expected that systemic environmental changes like climate change will have profound and complex effects on society [8,9], through the occurrence of extreme weather events and increased exposure to environmental hazards such as air pollution and disease [10,11]. Decision makers require the ability to evaluate the associated health risks in the context of public policy [12,13], however it is clear that few applications of EHIA have attempted to incorporate issues such as climate change [14]. To date, attempts have been made to inform the development of adaptation strategies [15], to assess the vulnerability of specific populations [16], and to directly assess both positive and negative impacts of climate change on health [17]. However, it has been noted that the methods required for the assessment of broad issues like climate change may be fundamentally different from traditional assessment methods [18].

It has been proposed that the effective incorporation of climate change-related health risks requires an integrated approach [19] that involves factors often omitted from the traditional assessment process [20]. These assessment methods have ranged from the purely mathematical approach of quantitative predictive scenario modelling [21,22], to the application of more generalised frameworks that attempt to account for complex interactions between the environment, health, related policies and activities, and the multi-disciplinary nature of the groups involved [19,23]. These generalised frameworks have identified as important not only quantitative aspects of health impacts but also “softer”, qualitative considerations that are often omitted from the traditional assessment process [20,24,25].

There are several problems associated with climate change that are relevant to the assessment process. Firstly, the issue of scale is important: given the complex nature of how climate change effects the environment [26], there are in turn many different pathways by which climate change can impact human health over different spatial and time scales [15]. The application of frameworks must also acknowledge that climate change and associated policies related to urban planning, energy and health have the potential to affect large and diverse sections of the population [27,28]. Therefore gaining input from various stakeholder groups is important to the assessment process [19,29,30,31]. Finally, how hazards are identified and measured can change due to non-stationary health effects [32], and examinations of such environmental–health linkages can be further complicated by a fragmented and uncoordinated approach to data collection [33].

It is unknown as to whether EHIA can adequately incorporate climate change-related health risks [4,34], and the practicalities associated with such an application have received limited attention. In order to answer these questions, the current study was conducted as part of a larger project, aimed at developing conceptual and practical approaches to the integrated and precautionary assessment of climate change-related health risks.

In this study, we used a focus group design to examine the views of experts from government, industry and academia on assessing the health consequences of climate change within an EHIA.

## 2. Experimental Section

### 2.1. Participants and Setting

A focus group design was implemented to evaluate the applicability of EHIA in the assessment of climate change-related health risks.

Two focus groups were conducted, each of which consisted of seven to 10 participants invited by email or phone. The participants were made up of representatives from relevant Queensland government departments such as the Departments of Environment and Resource Management (including the Queensland Climate Change Centre of Excellence), Transport and Health, Brisbane city council, along with academic and industry experts (Table 1). The participants were specifically selected due to their considerable experience in EHIA. Most had been extensively involved in environmental or health impact assessments ranging from the study of contaminant exposure in relation to industrial projects, the development of policy in relation to industry or health regulations, as well as health impact measurement associated with specific projects.

**Table 1:** Characteristics of focus group participants.

Specialists	Male	Female
Government-Environmental	8	2
Government-Health	2	0
Academia-Environmental	2	0
Academia-Health	1	1
Industry-Environmental	1	0

### 2.2. Focus Groups

The protocol for the focus groups was developed to explore the views of participants on the two main topics of interest: (i) the potential strengths and limitations of assessing the health consequences of climate change within an EHIA; and (ii) the practical issues associated with the integration and analysis of environmental and health data. Questions, created *a priori*, were intended to promote in-depth discussion on these issues. Table 2 lists the primary questions used to guide focus group discussions.

**Table 2:** List of the primary questions for focus groups’ discussion.

What are the major attributes of the current EHIA approach?
What are the strengths and weaknesses of the current EHIA, particularly in relation to large environmental changes?
As practitioners of EHIA, are you concerned about for example, sea-level rise?
Do you think stakeholder participation is handled adequately in the current EHIA framework?
How can we improve communication across different government departments, with the view to improve evidence-based decision making?
In your opinion, are current data sufficient for the purposes of EHIA, or are additional data required that are currently not collected?
How can data collection processes be improved?
How useful would it be to the EHIA approach to bring environmental and health data together in a single system?

Both sessions ran for 60 min and were recorded in audio format, copies of which were then transcribed verbatim and verified by the participants. Ethical approval was obtained from QUT’s Human Research Ethics Committee, and written informed consent was obtained from all participants in each focus group.

### 2.3. Design and Analysis

The data synthesis involved the application of Kruger’s framework method [35,36], and specific measures were employed in both the design (*i.e.*, purposive sampling, combination of inducting and deductive thematic design) and analysis (*i.e.*, multiple coders, theme refining) [37]. Transcripts were checked a number of times by two separate researchers, who separately collated main themes and sub-themes corresponding to the research questions [38,39]. Subsequent coding involved assigning text passages to a relevant theme/s, with the final framework then discussed in terms of definitions and boundaries of each theme, with any differences resolved.

## 3. Results and Discussion

Three main themes were identified (Table 3): attributes of the current EHIA framework, issues surrounding an EHIA of climate change, and the development of an environment and health information system.

**Table 3:** Main findings from focus group sessions.

Theme	Sub-theme
Attributes of the current EHIA framework	Frameworks have common structure
	Current framework has capacity for broad considerations
	Currently an established methodology to assess localised physiological exposure
	Facility to incorporate qualitative data
	Usually reliant on a legislative driver
Issues surrounding EHIA of climate change	Incorporation of a broad variety of issues
	The selection of appropriate health indicators
	Consideration of both positive and negative impacts
	Assessment of complex, multiple exposures and pathways
	Incorporation of socio-economic and other qualitative determinants of health
	The importance of appropriate stakeholder participation
The development of an environmental and health information system	Important to understand the purpose of the information system
	Linking of routine data is difficult (quality, suitability)
	Linking of data often forbidden (legal, political and confidentiality issues)
	Data is often dispersed (individual data silos)
	Need an appropriate assessment of data requirements

### 3.1. Attributes of the Current EHIA Framework

Participants identified various aspects of frameworks that are currently used to assess environmental exposure and related health impacts. There was some disagreement among the participants about whether a new or improved EHIA system is needed to assess climate change and its impact on health.

*“So do we have enough evidence that the existing frameworks are not working,* *thereby prompting you to attempt this new framework?”*

(Government-Environmental)

Participants agreed that the various frameworks for assessing impacts on health and the environment share a common structural capacity and that current assessment frameworks could be applied to a broad selection of different situations, provided that adequate assessment scope and boundaries were selected. It was agreed that the current frameworks could be suitable for the assessment of climate change and related health risks.

*“So if you were looking at say climate change in Brisbane or QLD then that would be part of your scoping and you would set for example a time frame,* *geographical location etc. In that way the HIA could lend itself to something broad”*

(Academia-Health)

This opinion was not unanimous, with some participants arguing that the current methodology was designed specifically for the assessment localized physiological exposure:

*“My feeling is that it will as it is just a standard logical sequence. The problem is that it is built for specific issues,* *like assessing cancer cases at the old ABC site. What we require here is something much more broadly based”*

(Academia-Environmental)

It was noted that in general, the application of such assessments is driven largely by associated legislative drivers; a factor that would be particularly important in relation to long term issues such as climate change.

*“Often it seems that proper implementation is reliant on a legislative driver, and without this it is done in an ad-hoc,* *case by case basis”*

(Academia-Health)

### 3.2. Issues Surrounding an EHIA of Climate Change

The discussions around the attributes of the current EHIA framework were then directed toward eliciting participants’ opinions regarding attributes that, if present, would make the framework amenable for use in assessing the health consequences of climate change. Several participants were of the view that complex environmental changes may be difficult to meaningfully assess, due to their nature and long time-scales. Concern was raised of the ability of assessment practitioners to incorporate the potentially large number and broad array of different climate change-related health risks. It was noted that this would have to be handled carefully to ensure the assessment process was manageable.

*“Climate change is difficult in itself to integrate, but the other aspects related to planning, how society is structured,* *how people live etc. are extremely broad topics to integrate into one single framework”*

(Government-Environmental)

Participants noted that the identification of appropriate health indicators and subsequent collection of environmental and other types of related data were vital to the assessment process. In terms of the actual selection of which indicators to use in a particular situation, it was noted that one factor to take into account in such a selection was that the choice of particular health indicators should be aligned with the issues being assessed.

*“I think it comes back to the basic point of knowing what the health indicators are and setting up databases,* *and I have the feeling that we’d be more successful in setting these things up if we knew what and where to monitor”*

(Government-Health)

It was also noted by some participants that in terms of implementation, it was necessary to be able to identify the exposure pathways between complex environmental changes and public health impacts. An example was given of issues related to the measurement of health impacts due to simultaneous exposure to multiple contaminants. The issue of understanding linkages is a research problem that in the participant’s view has not yet been dealt with:

*“We often do not know the linkage between health and the environmental indicators and which are important,* *and there are often lots of linkages we don’t capture”*

(Academia-Health)

In order for an EHIA to take a holistic view of all relevant issues, the scoping component of an assessment must allow for a broad analysis of both positive and negative impacts on health. Participants noted that it has previously been common for assessments to concentrate on negative impacts only:

*“Also, it is a question of how positive vs. negative impacts are considered; for example, how do changes in the environment benefit,* *or how changes may benefit some but disadvantage others. I’m not sure if that sort of analysis has been considered so far”*

(Academia-Health)

Socio-economic and other qualitative indicators (e.g., psychological stress) were identified as being important to provide a more complete picture of the relationship between health and climate change, and to allow the identification of vulnerable sections of the population. Although current the EHIA framework allows for the input of such data, it was suggested that this type of information may not be sufficiently utilised.

*“So in this way survey, discussions, and focus group sessions still produce valid information. It is challenging coming from a quantitative paradigm to grasp what this information means and how to analyse this type of data,* *but from a political perspective it is still important information, as often decisions are made not because there is a medical problem but because it is a perception problem”*

(Academia-Health)

The value of involving various stakeholders in decision making, and the collection of qualitative data relating to their concerns was emphasised, and it was thought that rarely is this involvement properly handled. It was noted that interviewing different stakeholder groups would enrich an assessment in terms of the selection of appropriate indicators and accessing data that otherwise may not be available. One participant illustrated the value of involving community stakeholders in the assessment process, noting a recent successful development:

*“This plant was up and running in a relatively short period of time, and the strength of the whole process was the way in which the community was involved—it was a brilliant job. No public relations process was used,* *all public consultation was done by the scientists and engineers involved in the plant’s construction, these people were involved and were open with the community. This resulted in the community wanting and embracing the project”*

(Government-Environmental)

Importantly however, appropriate levels of consultation are required to ensure that such stakeholder participation is effective, an issue that is sometimes badly handled as expressed by one participant:

*“I have seen projects where over-consultation has made the politicians involved nervous enough to pull out and the whole project collapses.* *So a lot of consultation can stop a project in a similar way as where insufficient consultation is judged as being morally corrupt”*

(Government-Environmental)

### 3.3. The Development of an Environment and Health Information System

One of the aims of the focus groups was to elicit stakeholders’ opinions about the usefulness and characteristics of a proposed environment and health information system. The system, implemented through a data portal accessible by decision makers, would facilitate the linkage of environmental, health and other related data using cutting-edge epidemiological techniques, and present the data in a form that would allow its use in assessment and policy related decision making. The feasibility of such an information system was discussed in terms of difficulties in connecting and making sense of large quantities of different data types. The participants noted that the needs of users would have to be understood and integrated during its development:

*“It has to be context specific….* *The database would have to have some specific constraints and users would in turn need basic knowledge of the data modelling and environmental health issues in order to use it in the way it’s meant to be used”*

(Academia-Health)

A general consensus was reached in both focus groups that the quality of available data is often a major issue. Problems surrounding the suitability of data were also raised as a barrier to effective data integration and utilization in the context of an EHIA. It was proposed that closer integration between environmental and health practitioners involved in an assessment was required, as often data appropriate for environmental assessment are either not collected or not useful for assessing health impacts. An example given was that of communicable disease notification data; collected for specific purposes and not amenable to epidemiological analysis to support impact assessment.

*“One thing that could be improved is that if a project is suspected to have a health impact at some point,* *then the question should be asked as to how we can design our environmental monitoring so that the data has more utility for health impact assessment”*

(Academic-Health)

Even when the appropriate data are collected, participants claimed that it is often difficult to share and use the data. This was due to legal and political reasons, along with physical and inter-departmental separation of data in disconnected locations. Participants noted that in collecting data, it was important to identify which data are collected routinely and which are collected on a case-by-case basis, and to note this difference when deciding how the data could be utilised in an assessment. Greater emphasis on collecting qualitative data in addition to quantitative information, and ensuring that stakeholders were involved throughout all stages of assessment were also listed as “must-do” strategies for enhancing the quality and application of data for the purpose of an EHIA.

To improve the applicability of such an information system, it was proposed that performing a data requirement assessment would be a useful exercise. This would result in an operations manual related to data collection, analysis and interpretation within the proposed methodology:

*“So thinking about those layers of health outcomes, what data is available, where does it come from and how do you access it, right down to ‘well the bottom line is that if you are examining this then you have to collect it,* *these are the tools and these are the limitations around those tools’. From a practitioners perspective this would be a useful thing to have, and I’m not sure something like that exists”*

(Academia-Health)

## 4. Conclusions

This study applied a focus group design to examine the views of government, academic, and private sector stakeholders on the current EHIA framework and its ability to incorporate climate change-related health risks. We were specifically interested in identifying those framework attributes that support such assessment, and those attributes that may require further development. We also examined views related to the development of an environment and health information system for evidence-based decision making. Participants generally agreed that while the current EHIA methods could incorporate the health consequences of climate change, the scope of assessment and selection of suitable health indicators would be important factors in such applications, with both concepts requiring further development in the existing framework. Discussions revealed the complexity of issues such as the logistics of collecting appropriate data and problems associated with confidentiality, as well as the feasibility of creating a single information system that would cater for the needs of a wide variety of end-users.

It has become increasingly recognised, particularly through the development of integrated assessment methods [4,19] that in order to assess the health consequences of climate change, EHIA must allow for a wider scope of applications and complex exposure pathways, and also take into account the increasing number of different disciplines and stakeholders that would be involved in the assessment process. The focus group discussions suggest that while specifically designed to assess localised issues surrounding developments and one-off projects, EHIA could currently incorporate aspects such as the scoping of a wider variety of environmental impacts, more effective stakeholder consultation, and the consideration of both positive and negative impacts of environmental determinants of health. However, it was noted that presently these issues were not developed to the extent that was needed to assess climate change-related health consequences.

For instance, participants noted that the assessment of positive impacts is often neglected in both conceptualisation and implementation stages of EHIA. It was revealed that while the current framework allows broad issues to be considered in the initial stages of assessment, the process itself is still based around measuring direct physiological exposure. This agrees with other findings that suggest that “softer”, qualitative issues, although important in regards to correctly framing the application in question, are rarely incorporated into the assessment process [40,41]. For example, psychological stress has often been a neglected effect of climate change [42,43], which can be impacted by many direct (loss of property or life due to flood damage) and indirect (implementation of related policies) issues.

In line with findings from the literature [13,14], our study identified stakeholder participation as vital to the assessment process. The complex nature of assessment requires input from those performing the assessment, those impacted and the decision makers who utilise the assessment findings. While climate change is a global issue involving large numbers of stakeholders, EHIA could only realistically target relevant groups that are exposed to localised effects or for which associated policy is relevant. These actors, due to the diverse nature of climate change, are often spread across many different disciplinary areas [15]. In our study participants were strongly of the view that stakeholder participation is often not well handled [44,45], with problems relating mainly to inadequate communication between stakeholder groups.

Finally, participants noted a number of implementation issues relating to both the conceptualisation of an assessment itself and problems surrounding data acquisition. The concept of an environment and health information system, providing an electronic platform through which environment, socio-economic and health data are linked using the latest epidemiological methods, could find application in several areas of impact assessment. Such a system inherently requires collecting data from a large number of different sources and as such, problems relating to access, availability and suitability of data can arise [16,46], and these issues are particularly relevant in regards to climate change. Similar observations have been made in other areas of public health [47], with the training of staff to interpret particular data, along with data ownership and data comparability identified as barriers to the use of a particular data source. In line with the literature [45,48], participants suggested that the process of collecting new data should be informed by the health indicators of interest in the assessment, and that the use of data collected for other purposes be informed by the purpose and nature of its initial collection. Communication between environmental and health workers could significantly improve the applicability of collected data, along with an initial data requirements analysis that would inform the collection itself.

This study has some limitations. Our review of current EHIA methods was limited, primarily due to the fact that consideration of health using an EHIA is still in a relatively early stage in Queensland, with most guidance related to the quantitative assessment of specific physiological exposures. Additionally, participant’s responses may have been biased as a result of holding differing ideas of EHIA process itself. Such variations could be resolved in future research by agreeing on a common framework structure prior to discussion. Our focus groups lacked substantial input from both local government and health authority practitioners, who in general may be more involved in planning and setting related environmental and health assessment requirements. Time constraints on the focus group sessions, along with cases of individuals dominating the sessions may also have prevented some participants from effectively expressing their views.

This study has identified several important issues that should be taken into account when developing and implementing an EHIA framework that incorporates and assesses the health risks of climate change. The study found that it is important to incorporate a broad range of exposure pathways and different types of quantitative and qualitative data, and to assess the risks in the wider context of the affected populations. In terms of the development of an environment and health information system to support evidence-based decision making, it is important that data collection is performed in a manner that takes into account the type of data, its context, confidentiality, relevance and practicality of access.

It is expected that these findings will aid in the improvement of existing EHIA methods that incorporate and assess the health risks of large scale issues like climate change that are of increasing concern to public health decision-makers. While the study was limited to Queensland, Australia, the results of this study may be applicable to the implementation of EHIA principles in the assessment of health consequences of systemic environmental change elsewhere.
